# Predictive Modeling for Frailty Conditions in Elderly People: Machine Learning Approaches

**DOI:** 10.2196/16678

**Published:** 2020-06-04

**Authors:** Adane Tarekegn, Fulvio Ricceri, Giuseppe Costa, Elisa Ferracin, Mario Giacobini

**Affiliations:** 1 Modeling and Data Science, Department of Mathematics University of Turin Turin Italy; 2 Department of Clinical and Biological Sciences University of Turin Turin Italy; 3 Unit of Epidemiology Regional Health Service, Local Health Unit Torino 3 Turin Italy; 4 Data Analysis and Modeling Unit Department of Veterinary Sciences University of Turin Turin Italy

**Keywords:** predictive modeling, frailty, machine learning, genetic programming, imbalanced dataset, elderly people, classification

## Abstract

**Background:**

Frailty is one of the most critical age-related conditions in older adults. It is often recognized as a syndrome of physiological decline in late life, characterized by a marked vulnerability to adverse health outcomes. A clear operational definition of frailty, however, has not been agreed so far. There is a wide range of studies on the detection of frailty and their association with mortality. Several of these studies have focused on the possible risk factors associated with frailty in the elderly population while predicting who will be at increased risk of frailty is still overlooked in clinical settings.

**Objective:**

The objective of our study was to develop predictive models for frailty conditions in older people using different machine learning methods based on a database of clinical characteristics and socioeconomic factors.

**Methods:**

An administrative health database containing 1,095,612 elderly people aged 65 or older with 58 input variables and 6 output variables was used. We first identify and define six problems/outputs as surrogates of frailty. We then resolve the imbalanced nature of the data through resampling process and a comparative study between the different machine learning (ML) algorithms – Artificial neural network (ANN), Genetic programming (GP), Support vector machines (SVM), Random Forest (RF), Logistic regression (LR) and Decision tree (DT) – was carried out. The performance of each model was evaluated using a separate unseen dataset.

**Results:**

Predicting mortality outcome has shown higher performance with ANN (TPR 0.81, TNR 0.76, accuracy 0.78, F1-score 0.79) and SVM (TPR 0.77, TNR 0.80, accuracy 0.79, F1-score 0.78) than predicting the other outcomes. On average, over the six problems, the DT classifier has shown the lowest accuracy, while other models (GP, LR, RF, ANN, and SVM) performed better. All models have shown lower accuracy in predicting an event of an emergency admission with red code than predicting fracture and disability. In predicting urgent hospitalization, only SVM achieved better performance (TPR 0.75, TNR 0.77, accuracy 0.73, F1-score 0.76) with the 10-fold cross validation compared with other models in all evaluation metrics.

**Conclusions:**

We developed machine learning models for predicting frailty conditions (mortality, urgent hospitalization, disability, fracture, and emergency admission). The results show that the prediction performance of machine learning models significantly varies from problem to problem in terms of different evaluation metrics. Through further improvement, the model that performs better can be used as a base for developing decision-support tools to improve early identification and prediction of frail older adults.

## Introduction

Health challenges associated with aging are a major medical and social concern as the burden of the older population is increasing dramatically. The elderly population, which has been conventionally defined as having a chronological age of 65 years or older [1], is becoming a meaningful challenge for every nation in terms of services and costs [2]. According to a 2017 United Nations report [3], the world population of older persons aged 60 years and above was 600 million in 2000, and it is projected to rise to approximately 2 billion by 2050. The aging of the population has profound consequences, with one of the main issues associated with this phenomenon being the higher prevalence of frailty condition [4]. Frailty is one of the most important and emerging age-related conditions that generally represents an increasing limitation in daily activities. Older people develop a wide variety of age-related conditions that contribute to an increase in their vulnerability to minor stressor events and lead to loss of autonomy. This phenomenon is commonly known as frailty [2,5]. People who are considered frail are particularly vulnerable to undesirable outcomes, including disability, injurious falls, hospitalization, and death. These health outcomes result in a poor quality of life and increased demand for medical and social care and are associated with increased costs for individuals and health systems. According to a study [6], health spending increases significantly in higher age classes compared with lower age groups. Older adults (those aged 70 years and older) are more likely to live with multiple chronic conditions and functional limitations. This combination is related to a larger probability of accessing an emergency department (ED) along with higher Medicare spending for inpatient hospitals, trained nursing facilities, and home health services. However, frailty is not an inevitable consequence of aging, and it can be prevented and managed to foster a longer and healthier life. Early detection and screening would help to deliver preventive interventions and reverse frailty conditions.

Several scales and models have been proposed for the detection of frailty [7-10]; however, a precise operational definition of frailty or a standard method for its screening and diagnosis is still lacking [11,12]. In clinical settings where the standard measure of frailty is missing and the care of the elderly is a priority, it is imperative to have a specific model in the prediction of frailty according to the characteristics of the population being studied. Therefore, this study aimed to detect multiple outcomes of frailty (mortality, disability, fracture, hospitalizations, and emergency admissions) using large administrative health databases on elderly people in Piedmont, Italy.

The study examines the existing machine learning techniques (artificial neural networks [ANNs], genetic programming [GP], support vector machines [SVMs], logistic regression [LR], decision trees [DTs] and random forests [RFs]) to predict frailty according to the different adverse health outcomes. These approaches were considered for their performance and practical usefulness in the analysis of different types of medical data.

## Methods

### Data Source

This study was based on the Piedmontese Longitudinal Study. The data were collected using an individual record linkage available for about 4 million Piedmont (Italy) inhabitants between the Italian 2011 census and the administrative and health databases (enrollees registry, hospital discharges, drug prescriptions, outpatient clinical investigation database, and health exemptions) that is included in the Italian Statistical National Plan. Subjects aged 65 years and above are included in the study. The dataset contains 1,095,612 subjects and 64 variables (58 input and 6 output variables). The dataset includes a wide variety of predictor variables, including clinical and socioeconomic aspects, and six target variables for every subject: mortality, disability, urgent hospitalization, fracture, preventable hospitalization, and accessing the emergency department (ED) with red code. Color codes assigned to patients may vary from one hospital to another, but in this study, a red code is used to identify patients with severe symptoms who need an immediate care. Since we intend to develop predictive models for these frailty indicators, we extracted as input data those collected in 2016, while using as output values those collected in 2017.

For simple implementation and analysis, the data were transformed into six datasets, one for each output variable. As a result, six problems associated with frailty conditions were identified and defined. The six datasets were considered separately in the analysis, which resulted in six independent binary classification problems. All the input variables used in the study are presented in Multimedia Appendix 1. Table 1 contains descriptive statistics for all output variables with the frequency distributions of each category of an output variable represented as counts and percentages. Table 1 clearly shows how the dataset is, for each output variable, unbalanced. In fact, approximately 4% of the records have mortality risk as 1, and the other 96% have mortality risk as 0. There are similar numbers of records having risk as 1 for emergency admission with red code, fracture, preventable hospitalization, disability, and urgent hospitalization. This is clearly an indication of an imbalanced dataset, as the number of subjects from the positive sample is much smaller than the number of subjects of the negative sample.

**Table 1:** Description of output variables in the dataset.

Variable		Code	Value, n (%)
**Mortality**			
	No	0	1,053,790 (96.18)
	Yes	1	41,823 (3.82)
**Accessing the ED^a^ with red code**			
	No	0	1,088,124 (99.32)
	Yes	1	7489 (0.68)
**Disability**			
	No	0	1,064,186 (97.13)
	Yes	1	31,427 (2.87)
**Fracture**			
	No	0	1,088,530 (99.35)
	Yes	1	7083 (0.65)
**Urgent hospitalization**			
	No	0	1,056,695 (96.45)
	Yes	1	38,918 (3.55)
**Preventable hospitalization**			
	No	0	1,076,541 (98.26)
	Yes	1	19,072 (1.74)

**^a^**ED: emergency department.

Most machine learning techniques suffer from such extremely unbalanced datasets, and, as a result, they may be biased toward the majority class. Instructing a model with an algorithm that tries to maximize the accuracy will naturally lead to classifying everything as the major class and will not give acceptable results.

### Handling Imbalanced Dataset

The dataset in each problem (mortality, accessing ED with red code, disability, fracture, urgent hospitalization, and preventable hospitalization) is imbalanced, as shown in Table 1. The imbalanced proportions between the positive and negative classes of the six datasets are treated independently. There are various approaches to deal with imbalanced data that have been used in the literature, such as resampling [13] and cost-sensitive learning methods [14].

In this study, we chose the resampling methods, which are based on undersampling [15] and oversampling [16]. These methods are advantageous because they are classifier independent and can be used as a preprocessing step, in which the processed data can be given as input to any classifier. Oversampling is the process of replicating samples from the minority class to balance the data. The limitation of oversampling is that it may cause an overfitting problem as it clones the same instance and requires more time to execute compared with the undersampling approach. As a result, it is recommended when the dataset is quite small in size. Another issue with oversampling is that as our aim was to detect minority classes, oversampling changes the class that we want to identify, which may not be acceptable in some critical real-time problems [17]. Undersampling balances the imbalanced data by reducing the size of samples from the majority class. One limitation of the undersampling approach is that it may lead to loss of important information or introduce bias in the data. From a practical point of view, some literature showed that undersampling tends to outperform oversampling in some settings [18], while others demonstrate that oversampling performs better than undersampling [19]. In high-dimensional data, oversampling performs worse [20], while undersampling performs worse in very small datasets. In our case, since the amount of collected data is sufficient, we adopted undersampling to rebalance the sample distribution followed by a statistical test to avoid bias and ensure representativeness between samples. Since we have multioutput data, we followed these simple steps to obtain balanced and independent datasets:

- Filter all positive and negative samples from the original dataset based on the values of the output variables. Samples with at least one positive class value from the six outcomes are grouped as a positive sample, which accounts for 10% of the original dataset, and all remaining are grouped as a negative sample, comprising 90% of the original dataset.
- Keeping all the 10% samples in the positive class (minority group), we randomly selected an equal number of samples (10%) from the negative class (majority group).
- Check whether the randomly selected 10% negative samples were representative of the remaining negative samples (90%). After checking that the test was reasonably significant, we obtained a new multioutput dataset of size 211,924 each. A statistical test was applied in all variables to decide whether the distribution of frequencies of a variable in the 10% sample was representative of the same variable in the 90% sample. Since all variables in the study are categorical, we used a chi-square independence test with a significance level of .05 to check if there was a significant difference between the 10% sample and the 90% sample with respect to input variables. The yielded chi-square statistic and *P* values were assessed to support the significance of the test’s conclusion. The results of the chi-square test between 10% and 90% negative samples are shown in Multimedia Appendix 1.
- Once the test was significant, we decomposed the multioutput dataset into six independent datasets. An equal number of positive and negative samples were then selected randomly from each dataset.

### Predictive Models

The machine learning approaches selected for this study are SVMs, ANNs, RFs, DTs, LR, and GP. We have presented below a brief summary of these learning algorithms.

SVM is a robust classifier to identify two classes that require a huge amount of training data to select an effective decision boundary. Several studies have used SVM on disease prediction [21-24]. The SVM algorithm is used to predict events by plotting the training dataset where a hyperplane classifies the points into two classes, presence and absence of frailty. SVM is based on kernel functions, which project linearly inseparable input data to higher dimensional space for better classification. Various kernels and parameters are used to improve the performance of classification by SVM [25]. In this study, the radial basis function kernel is used with different values of gamma and the regularization parameters for solving each classification problem.

ANNs are analytical techniques that have been successful in solving classification problems in different domains [26-30]. Based on the functioning of biological neural networks, ANNs are dense networks of interconnected artificial neurons that get activated based on inputs. The multilayer perceptron neural network (MLPNN), one of the most used paradigms in ANNs, is employed in this study. The MLPNN includes one input layer, one or more hidden layers, and one output layer. In MLPNN, the input nodes pass values to the first hidden layer, and the nodes of the first hidden layer pass values to the second layer and so on, until producing outputs. The main parameters used in MLPNN, including activation function, solver, hidden layer size, and learning rate, are configured for each classification work.

We also explored the potential of tree-based classifiers (DTs and RFs) for the prediction of outcomes in each frailty problem. DTs build classification models in the form of a tree structure [31]. The main algorithms used in DTs are ID3, C4.5, and the classification and regression tree [32], which build DTs using the concept of information entropy. In our study, the classification and regression tree algorithm is used for building the DT with hyperparameters set for each problem. RFs consist of a large number of individual DTs that operate as an ensemble. Each tree gives a classification, and the forest chooses the classification having the most votes (over all the trees in the forest). RF is known for the prediction task in the medical domain [33-35]. The hyperparameters (such as the number of trees in the forest, maximum number of features considered for splitting a node, maximum number of levels in each DT, etc) have been set for each problem.

LR, a specific type of multivariate regression, is the most common and well-established binary classifier [36]. LR is used to model only a dichotomous variable, which usually represents the presence or absence of an outcome or event based on a set of predictor variables. It predicts an event of occurrence by fitting a dataset into a logit function. In this study, like other machine learning models, LR has been used to distinguish frail and nonfrail subjects.

Another technique applied to the prediction task is GP, typically designed to address the problem of automatic program synthesis and automatic programming. GP accomplishes this task by generating a population of computer programs over many generations using operations of natural selection [37]. Many works in GP focus on classifier induction, a task that can be accomplished by evolution using GP [38,39]. In GP, setting the control parameters is an important first step to manipulate data and obtain good results. In our datasets, we tried several experiments for classification tasks by using the control parameters of GP proposed in HeuristicLab tools [40]. The parameter values of GP used for our experiment are listed in Multimedia Appendix 2.

### Performance Metrics

**Figure 1:**
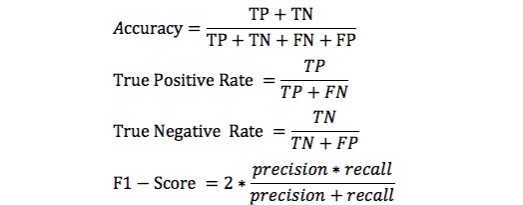
Evaluation metrics.

The performance measures were considered based on the proportion of older people with mortality, urgent hospitalization, preventable hospitalization, disability, fracture, and ED admission with a red code. Predicting these adverse outcomes among a large number of subjects is important when applied in real-world practice. Hence, the true positive rate (TPR) was the main metric to consider. The overall accuracy, true negative rate (TNR), and F1-score, which is the harmonic mean of precision and recall, were used as additional performance metrics. The accuracy, TPR, and TNR were formulated using the true positives (TPs), false positives (FPs), true negatives (TNs), and false negatives (FNs). These measures are defined in the equations in Figure 1 [41].

### Data Analysis Tools

The data analysis tools used in the study are Python Scikit-learn library, RStudio software package, and HeuristicLab. In this work, the exploratory data analysis part and statistical test analysis were done using R3.5.0, whereas the entire classification problems with SVMs, RFs, NNs, and DTs were implemented using Python 3.7. Multimedia Appendix 3 presents some Python codes used in the experiment. HeuristicLab is a software tool for heuristic and evolutionary algorithms. In this study, HeuristicLab was used to carry out classification problems using GP.

### Experimental Settings

#### Model Evaluation

**Figure 2:**
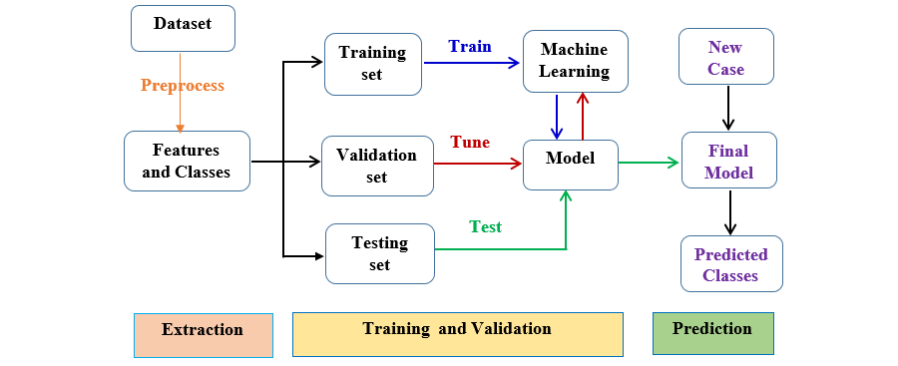
Experimental workflow of the predictive machine learning model.

In analyzing the data for prediction, the output variables represent an occurrence in the next year, and the predictive model is proposed to predict frailty according to the expected risk of urgent hospitalization, preventive hospitalization, disability, fracture, accessing the ED with a red code, and death within a year. The performance of various predictive models is evaluated for each outcome prediction using four metrics: accuracy, TPR, TNR, and F1-score. These metrics provide an effective and simple way to evaluate the performance of a classifier. Using these four measures, the models were evaluated using both the holdout method [42] and the cross-validation method [43]. Figure 2 shows the general experimental workflow of the predictive machine learning model.

#### Holdout Method

In this study, our first experiment was started by exploring the predictive performance of machine learning methods using the holdout method. This method randomly splits a dataset into training and testing according to a given proportion. Each machine learning model was trained using the training dataset (70%) and evaluated using test datasets (30%). The training dataset was used for building the model, while the test dataset was used to evaluate the prediction capabilities of models.

#### K-Fold Cross-Validation

The K-fold cross-validation procedure was applied to each problem’s data. Cross-validation is one of the most commonly used model evaluation procedure that extends the holdout method by repeating the splitting process several times. The K-fold cross-validation technique divides the dataset into K folds of roughly equal size. The model being evaluated is then trained using the K-1 parts, and one part is left out for model validation. In this study, we used 10-folds, and the dataset was split into three parts for the purpose of model training and testing: the training set to build the model, validation set to select the model parameters, and test set to evaluate the performance of the final model based on the selected parameters.

#### Hyperparameter Tuning

In all experiments, the set of hyperparameters was selected for each machine learning method before the training begins. Hyperparameters allow machine learning algorithms to better adjust to the problem details. The hyperparameters for each model were tuned using a grid search with cross-validation in Python Scikit-learn as described by Mueller and Guido [44]. Multimedia Appendix 2 presents the list of hyperparameters used for training each machine learning model in this study.

## Results

### Study Population

From the original dataset of 1,095,612 elderly people aged 65 years and above, we retrieved 83,646 with mortality, 77,836 with urgent hospitalization, 62,854 with disability, 38,144 with preventable hospitalization, 14,978 who accessed the ED with red code, and 14,166 with a fracture for this study. The retrieval process was made using the resampling approach, and each problem was analyzed independently of the others using the widely used machine learning models. In this section, the predictive performance of machine learning models using both holdout and cross-validation methods are presented through feature selection analysis.

### Feature Selection

Feature selection provides an effective way to remove irrelevant and/or redundant features, which can reduce running time, increase learning accuracy, and facilitate a better understanding of the model [45,46]. Unnecessary features can also increase the chance of overfitting and decrease the generalization performance on the test data. We used a filter method for feature selection [47,48]. A chi-square test is a filter method used in this study to determine the statistical significance between features and the target. The chi-square value, together with *P* values at a significance level of .05, was used to identify the most important features with their rank (ie, variables shown to be significantly associated with the outcome by the chi-square test analysis [*P*<.05] were selected for model building). *P*<.001 indicates that there is an association between the input and the target variables. The strength of the association between the input variables and the target is ranked based on the chi-square value. Out of the 58 predictor variables, 25, 24, 10, 7, 4, and 3 nonsignificant variables were discarded for preventable hospitalization, urgent hospitalization, emergency admission with red code, fracture, mortality, and disability, respectively. Table 2 presents the top 15 ranked features in order of decreasing importance in the mortality and fracture problems. The most significant feature for other problems is presented in Multimedia Appendix 4.

**Table 2:** The most important variables in the mortality and fracture problems.

Rank	Mortality problem		Fracture problem	
	Variable	*P* value	Variable	*P* value
1	Age	<.001	Age	<.001
2	Charlson index	<.001	Femur fracture	<.001
3	# urgent hospitalization	<.001	# urgent hospitalization	<.001
4	# total hospitalization	<.001	Neck fracture	<.001
5	Invalidity	<.001	Green code	<.001
6	# nontraumatic	<.001	# total hospitalization	<.001
7	Disability	<.001	Charlson index	<.001
8	Poly prescriptions	<.001	Poly prescriptions	<.001
9	Green code	<.001	Invalidity	<.001
10	Yellow code	<.001	Disability	<.001
11	Blood	<.001	Nerve disease	<.001
12	Anemia	<.001	Depression	<.001
13	Circulatory disease	<.001	Blood	<.001
14	Respiratory disease	<.001	Anemia	<.001
15	Urinary tract disease	<.001	Yellow code	<.001

Feature importance can give us insight into a problem by indicating what variables are the most discriminating between classes. For example, in Table 2, age and the Charlson index are the most important features in the prediction of mortality, which makes sense in the problem context. The rank of features differs from one problem to another, except for the variable age, which has the highest score in all problems. Next to the age attribute, variables such as femur fracture, number of urgent hospitalizations, and neck fracture are the most discriminant features in the fracture problem, while type of family and home living status are the least significant variables. Mental disease, poly prescription, and disease of the circulatory system are variables with the highest rank in urgent hospitalization and preventable hospitalization. The age, Charlson index, and number of urgent hospitalizations are the most important predictors of emergency admission with red code. Some features with the lowest rank and common to urgent hospitalization and preventable hospitalization include marital status, level of education, work status, and income. Each of the predictive models (SVM, ANN, LR, RF, and DT) have been applied using the most important features in each of the six problems. GP differs from the other machine learning models in that it performs implicit feature selection automatically during the evolutionary process. GP learns which combination of features are useful for classification and determines the optimal number of features automatically.

### Performance via Holdout Method

**Figure 3:**
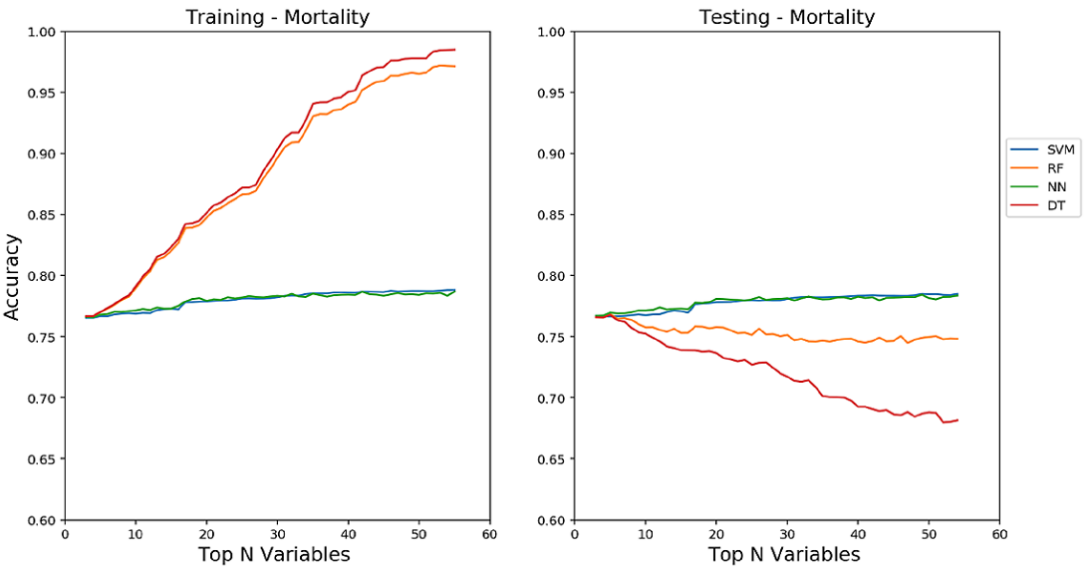
Train accuracy (left) and test accuracy (right) for mortality data without performing any parameter tuning and using all the feature subsets (from top 3 to top 58 feature subsets). The left plot shows that random forest and decision tree overfit the training data, which poorly generalize on the test data as the number of features increase.

**Figure 4:**
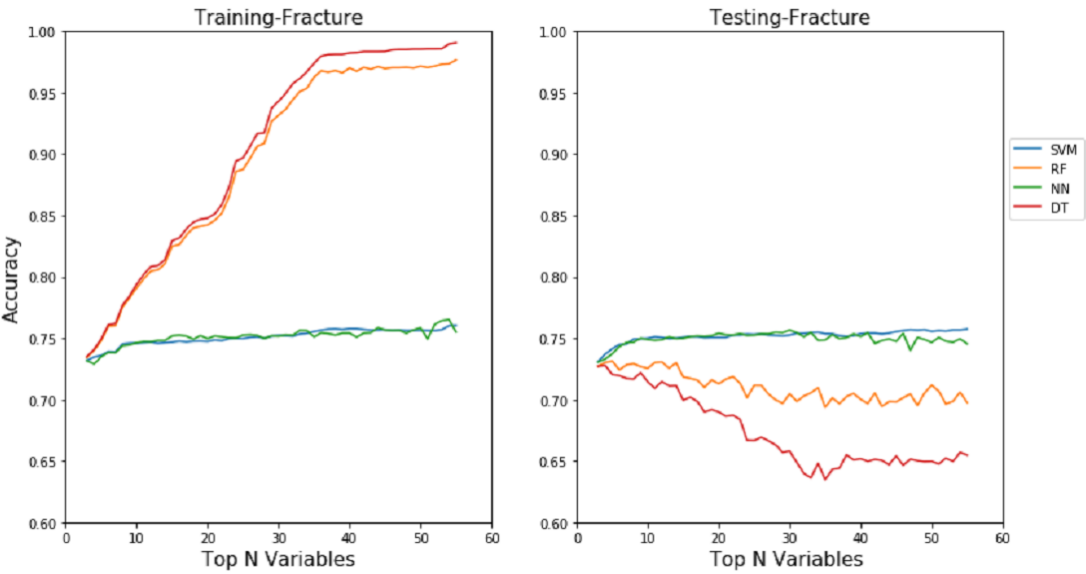
Train accuracy (left) and test accuracy (right) for fracture data without performing parameter tuning and using all the feature subsets (from top 3 to top 58 feature subsets). The left plot shows that random forest and decision tree overfit the training data, which poorly generalize on the test data as the number of features increase.

In this study, our first experimental results were obtained through the holdout (train-test split) method with all subsets of features (from top 3 to top 58 features) using the default parameters of the models. However, these approaches have brought the problem of overfitting on the training data for RF and DT, as shown in Figures 3 and 4. In order to reduce the overfitting problem and improve performance, the parameters of each model were tuned using grid search along with the most important features associated with each outcome. Table 3 shows the performance of SVM, RF, ANN, DT, and GP using the best features and parameters selected on each problem.

**Table 3:** Prediction performance using true positive rate and true negative rate for the six problems.

Problem	SVM^a^			RF^b^			ANN^c^			DT^d^			GP^e^	
	TPR^f^	TNR^g^	TPR		TNR	TPR		TNR	TPR		TNR	TPR		TNR
Mortality	0.78	0.78	0.79		0.77	0.79		0.78	0.60		0.79	0.75		0.76
Disability	0.78	0.72	0.78		0.71	0.75		0.75	0.78		0.69	0.71		0.67
Fracture	0.75	0.74	0.77		0.72	0.77		0.72	0.79		0.66	0.70		0.73
Urgent hospitalization	0.61	0.73	0.65		0.68	0.66		0.68	0.64		0.68	0.66		0.62
Preventable hospitalization	0.74	0.73	0.73		0.72	0.73		0.73	0.76		0.66	0.73		0.64
ED admission^h,i^	0.63	0.73	0.63		0.72	0.63		0.74	0.62		0.73	0.73		0.63

^a^SVM: support vector machine.

^b^RF: random forest.

^c^ANN: artificial neural network.

^d^DT: decision tree.

^e^GP: genetic programming.

^f^TPR: true positive rate.

^g^TNR: true negative rate.

^h^ED: emergency department.

^i^with a red code.

In our experiments, we explored common variations for each machine learning algorithm in frailty predictions. From the results of the experiment in Table 3, it is clear that all algorithms behave differently for each different problem. For the mortality dataset, RF and ANN produced higher values of TPR (0.79) while the DT produced the lowest performance. For the fracture problem, DT scored the highest values of TPR (0.79), while GP scored the lowest value. GP, on the other hand, has higher values of TPR on the urgent hospitalization dataset. The overall average TPR of RF was slightly higher for all problems, while SVM has slightly higher values of TNR in all problems and DT produced the lowest average TPR in all problems. According to the results on the test part of the dataset, all machine learning models showed lower prediction performance on the urgent hospitalization and accessing the ED with red code problems, while mortality and disability have higher values of prediction results compared with other outcomes. On the disability problem, GP has lower TPR compared with SVM, RF, ANN, and DT, while it has the highest TPR on accessing the ED with red code. For other problems, GP produces comparable results. The performance of GP is compared with other machine learning methods using statistical tests to draw better conclusions. We performed a pairwise statistical test between the 30 runs of GP and each individual machine learning model using the Wilcoxon signed-rank test. The Wilcoxon statistical test is a nonparametric test that ranks the differences in performances of GP and other algorithms over each frailty problem. The Wilcoxon test is based on the TPR of each algorithm in each problem on the test data. The results of the test in terms of *P* values with the significance level of .01 are shown in Table 4.

**Table 4:** Results of Wilcoxon signed-rank test in terms of *P* values.

Problem/dataset	SVM^a^ vs GP^b^	RF^c^ vs GP	NN^d^ vs GP	DT^e^ vs GP
Mortality	<.001	.003	.001	<.001
Fracture	<.001	.02	<.001	.002
Disability	.06	.004	.01	.003
Urgent hospitalization	.71	.01	.37	.01
Preventable hospitalization	.68	.03	.87	.005
Accessing the ED^f^ with a red code	.006	<.001	.01	<.001

^a^SVM: support vector machine.

^b^GP: genetic programming.

^c^RF: random forest.

^d^NN: neural network.

^e^DT: decision tree.

^f^ED: emergency department.

As depicted in Table 4, the Wilcoxon test allows rejecting 11 hypotheses. The *P* values below .01 indicate that the respective algorithms differ signiﬁcantly in TPR, while the *P* values above .01 indicate that the algorithms behave similarly in predicting frailty conditions. The test results between SM and GP are statistically signiﬁcant only in disability, urgent hospitalization, and preventable hospitalization. Combining the experimental results and Wilcoxon signed-rank test results, it is concluded that for mortality and fracture SVM outperformed GP in the TPR score, while GP outperformed SVM and RF on urgent hospitalization and accessing the ED with red code. Despite the fact that DT represented higher values of TPR on the preventable hospitalization compared with other algorithms, its lowest TNR result represented a higher disadvantage. ANN has a similar performance with GP for preventable and urgent hospitalization events.

### Performance via 10-Fold Cross-Validation

**Figure 5:**
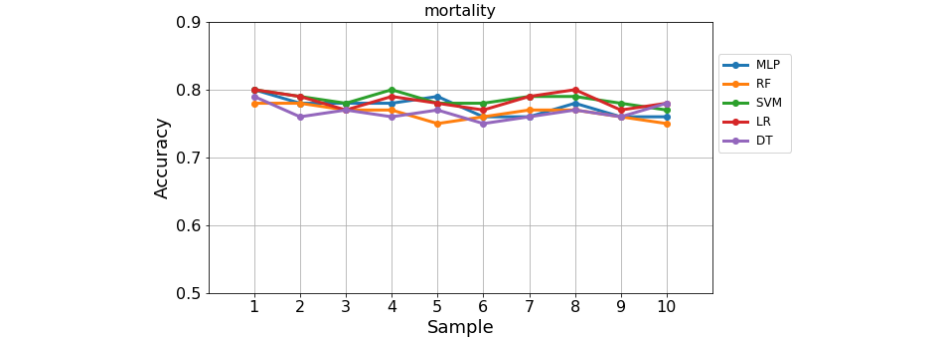
The score of five models across 10 validation samples on the mortality problem.

**Figure 6:**
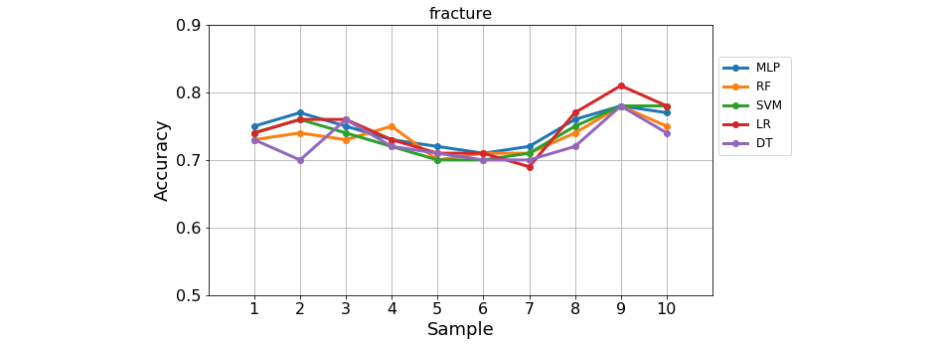
The score of five models across 10 validation samples on the fracture problem.

The 10-fold cross-validation reduces the variance of the resulting estimate by averaging over 10 different subsamples. This 10-fold cross-validation can deal with limitations of the holdout method, such as to reduce overfitting, and therefore is more reliable and provides better generalization performance on the test data. Thus, in our second experiment, we used the 10-fold cross-validation method on each of the six datasets. The variation of each model’s accuracy across the 10 samples in the 10-fold cross-validation is presented in Figures 5 and 6 for the largest dataset (ie, mortality) and smallest dataset (ie, fracture), respectively. From the figures, one can see that the models are more stable in predicting mortality than fracture across the 10 samples. It is also found a slight variation of classification rate across the 10 samples for the other outcomes.

As shown from Figure 5, the classification rate across 10 samples in the 10-fold cross-validation is slightly varied in each classifier for the mortality problem. The variation of accuracy is greater in the fracture problem from sample 1 to sample 10 for each model, as shown in Figure 6. Particularly, LR has shown the greatest variation of performance among the models, where it performed the lowest accuracy at sample 7 and the highest accuracy at sample 9 in the fracture problem. DT has shown the highest classification rate at sample 10 for mortality and at sample 3 in the fracture problem, while it has the lowest accuracy in the rest of the samples. The average performance of 10-fold cross-validation in each problem is shown in Table 5, where performance for each model is measured using accuracy, TPR, TNR, and F1-score.

**Table 5:** Prediction results of models using a 10-fold cross-validation.

Models		Accuracy	TPR^a^	TNR^b^	F1-score
**Mortality**					
	ANN^c^	0.78	0.81	0.76	0.79
	SVM^d^	0.79	0.77	0.80	0.78
	RF^e^	0.78	0.79	0.76	0.76
	LR^f^	0.78	0.78	0.79	0.78
	DT^g^	0.75	0.80	0.70	0.76
**Fracture**					
	ANN	0.75	0.77	0.73	0.75
	SVM	0.75	0.77	0.74	0.75
	RF	0.75	0.78	0.72	0.76
	LR	0.75	0.75	0.75	0.75
	DT	0.74	0.76	0.72	0.74
**Disability**					
	ANN	0.74	0.76	0.71	0.75
	SVM	0.75	0.78	0.73	0.76
	RF	0.75	0.77	0.72	0.75
	LR	0.75	0.76	0.73	0.74
	DT	0.73	0.78	0.70	0.75

^a^TPR: true positive rate.

^b^TNR: true negative rate.

^c^ANN: artificial neural network

^d^SVM: support vector machine.

^e^RF: random forest.

^f^LR: logistic regression.

^g^DT: decision tree.

From the results of all models in each outcome presented in Tables 5 and 6, we can see that predicting mortality events has shown the highest performance, while predicting urgent hospitalization and accessing the ED with red code have shown lower performance. Next to the mortality problem, better prediction performance is obtained on disability and fracture problems. This implies that the dataset in this study is better at predicting mortality than predicting the other outcomes. In predicting urgent hospitalization, only SVM achieved the best performing algorithm in all measurements (accuracy, TPR, TNR, and F1-score) among all models trained using 10-fold cross-validation. In the mortality problem, the highest average performance was obtained by ANN (accuracy 0.78, TPR 0.81, TNR 0.76, F1-score 0.79) and SVM (accuracy 0.79, TPR 0.77, TNR 0.80, F1-score 0.78) followed by LR (accuracy 0.78, TPR 0.78, TNR 0.79, F1-score 0.78). DT produced the highest TPR (0.80), and RF showed comparable results (accuracy 0.78, TPR 0.79, TNR 0.76, F1-score 0.76) on the mortality problem. For the fracture and disability problems, SVM, RF, and LR have similar accuracy (0.75), although they all differ in TPR, TNR, and F1-score.

**Table 6:** Prediction results of models using a 10-fold cross-validation procedure.

Models		Accuracy	TPR^a^	TNR^b^	F1-score
**Urgent hospitalization**					
	ANN^c^	0.67	0.64	0.71	0.66
	SVM^d^	0.75	0.77	0.73	0.76
	RF^e^	0.66	0.65	0.67	0.66
	LR^f^	0.67	0.72	0.62	0.65
	DT^g^	0.66	0.65	0.67	0.65
**Preventable hospitalization**					
	ANN	0.74	0.73	0.74	0.73
	SVM	0.74	0.71	0.76	0.73
	RF	0.73	0.73	0.74	0.73
	LR	0.74	0.71	0.76	0.73
	DT	0.72	0.73	0.71	0.72
**Accessing the ED^h^ with red code**					
	ANN	0.70	0.65	0.74	0.67
	SVM	0.68	0.64	0.72	0.66
	RF	0.68	0.66	0.70	0.67
	LR	0.69	0.64	0.74	0.67
	DT	0.67	0.70	0.65	0.68

^a^TPR: true positive rate.

^b^TNR: true negative rate.

^c^ANN: artificial neural network.

^d^SVM: support vector machine.

^e^RF: random forest.

^f^LR: logistic regression.

^g^DT: decision tree.

^h^ED: emergency department.

From the results of the experiments, it is important to observe that the various machine learning techniques can significantly vary in terms of their performance for the different evaluation metrics. For example, in the mortality problem, SVM outperformed DT and ANN in TNR value (.80), and ANN outperformed both SVM and DT in F1-score (0.79), while DT outperformed both models in TPR value (0.80). The performance of all models differs in all problems due to the difference in feature space, size, and diversity of data in each of the six problems. The prediction performance of all models trained with mortality data (largest in size) is much better than the performance of models trained with accessing the ED with red code data (smaller in size), which demonstrates that the size of data is an important factor for better performance, but this is not always true for all models. In addition, the performance of each machine learning technique varied from problem to problem. For example, the performance of ANN measured in TPR is 0.81, 0.77, 0.76, 0.74, 0.70, and 0.67 for mortality, fracture, disability, preventable hospitalization, accessing the ED with red code, and urgent hospitalization, respectively, while for DT the TPR is 0.80, 0.75, 0.78, 0.73, 0.70, and 0.65 for each problem, respectively. Considering the performance of these two machine learning methods (ANN and DT) in their TPR value, ANN outperforms DT in mortality and fracture problems, while DT outperforms ANN in disability and accessing the ED with red code problems. We can also see that LR has a higher TPR value than SVM in the mortality problem. This shows that it is not necessarily true that the more complex machine learning models (eg, ANN, SVM) always outperform simpler models (eg, DT, LR). In 10-fold cross-validation, the RF classifiers achieved comparable performance to SVM and ANN in most of the problems. On the other hand, tree-based classifiers (RF and DT) are more sensitive to bad features and quality of data. Therefore, effective feature selection is an important step to improve their performance. The SVM model tends to perform well in high-dimensional classification problems; however, it may not perform well if the sample classes of the problem are highly overlapping. ANN can generally outperform other techniques if the dataset is very large and if the structure of the dataset is complex (eg, if it has many layers).

In general, machine learning is an exploratory process, where there is no one-size-fits-all problem. In particular, there is no model recognized to achieve supreme performance for all problem types, domains, or datasets [49]. The best performing machine learning model differs from one problem to another according to the characteristics of variables, size of the data, and metrics used. The idea is similar to the “no free lunch” theorem [50,51], which states that there is no universal algorithm that works best for every problem. However, it is important to study each problem by evaluating each model carefully in order to reach an effective predictive design. The results also show it is essential to carefully explore and evaluate the performance of machine learning techniques using various optimized parameter values as well as using the most significant predictor variables. Particularly, tree-based classifiers (eg, RF and DT) are more sensitive to overfitting problems, as shown in Figures 3 and 4 on the mortality and fracture problems, if the correct subset of features is not selected or if the required parameter values of models are not configured properly. The accuracy in the figures clearly indicates that an increasing number of features in RF and DT leads to the model overfitting. Interestingly, SVM and ANN models showed relatively consistent performance both on training and testing even with an increasing number of features.

## Discussion

### Principal Findings

A predictive model that can use administrative health data will be useful in various settings to classify those individuals who are at risk of frailty and deliver preventive interventions. In this study, we performed several experiments using different classification techniques to build predictive models for frailty. The results show that machine learning models can vary significantly from problem to problem in terms of different evaluation metrics. The explored models have shown solid predictive power to better estimate the risk of mortality than predicting disability, fracture, emergency admission in red code, urgent hospitalization, and preventable hospitalization within the next year. Although each model is not a comprehensive model to predict all frailty outcomes, we have demonstrated that the SVM model has shown higher overall accuracy (0.79) in predicting mortality and urgent hospitalization than other models, when using 10-fold cross-validation. On the other hand, except for the ANN, all other machine learning models have shown relatively poor overall accuracy in predicting emergency admission with red code.

In addition, our results show significant performance enhancement by reducing features. In order to reduce the overfitting problem and improve the prediction performance of classifiers, the feature selection process is executed, where the best subset of the available features is chosen. In each binary classification problem, all independent variables were ranked using the chi-square feature selection method for each outcome in both holdout and cross-validation methods. Using 10-fold cross-validation on mortality problems, the TPR values (also called sensitivity) of ANN, SVM, RF, LR, and DT were 0.81, 0.77, 0.79, 0.78, and 0.80, respectively. In the holdout method, almost similar results were obtained for ANN, SVM, and RF, while DT produced higher TPR values using 10-fold cross-validation than holdout method on the mortality problem. In general, 10-fold cross-validation reduces variance by averaging over 10 different partitions; it is then less sensitive to any of the partitioning bias in the training and testing data. On predicting emergency admission with red code, GP achieved better TPR value than SVM, ANN, LR, RF, and DT, while SVM outperformed all models in predicting urgent hospitalization in all evaluation measures.

Generally, an important observation from the results of the experiments is that on average some of the machine learning models produce quite similar results from the same outcome, while the best performing model varies from one outcome to another outcome in terms of different metrics. For example, SVM and ANN produce similar performance on average across all evaluation metrics in mortality and hospitalization outcomes. RF and LR produced similar performance on average across all measurements in disability and fracture outcomes. However, the prediction results of each machine learning model varies from mortality to fracture or fracture to hospitalization, etc. This can demonstrate the feasibility of identifying frail older subjects through routinely collected administrative health databases.

### Strengths and Limitations

The strength of our study is the possibility to include a multidimensional administrative database using the most powerful predictive machine learning models. In contrast to the previous studies, the prediction models use a wide variety of input variables, including clinical and socioeconomic aspects, with six simultaneous outcomes. The use of routinely collected socioclinical data can represent the multidimensional loss of an individual’s reserves, which allows predicting prospective outcomes in the elderly. Moreover, the predictions of frailty in terms of the six adverse outcomes were assessed and analyzed, which is a step forward in studying the association of frailty with multiple health conditions on a frail person.

There are limitations to our study. Even though the original data comes with multiple outcomes, each machine learning algorithm was designed to predict a single outcome, and each result is analyzed independently of the others. Therefore, further studies should investigated constructing a predictive model that considers the correlations among the output variables to provide a list of relevant outputs for a given, previously unseen patient. Furthermore, patient information such as gender can be included in the study in order to understand gender-related factors for frailty and their impact on hospitalization and mortality among older people.

### Conclusions

Predictive modeling using the information available from administrative health databases is an efficient method to identify frail older people appropriate for interventions to prevent adverse outcomes. The proposed predictive models can be applied to detect and predict frail people who are at increased risk of adverse outcomes. This study suggests that a machine learning–based predictive model could be used to screen future frailty conditions using clinical and socioeconomic variables, which are commonly collected in community health care institutions. With efforts to enhance predictive performance, such a machine learning–based approach can further contribute to the improvement of frailty interventions in the elderly community.

## Appendix

Multimedia Appendix 1 Description of variables and statistical test between samples.

Multimedia Appendix 2 Hyperparameters used for training models.

Multimedia Appendix 3 Python implementation codes used in the study.

Multimedia Appendix 4 List of most important features in each outcome.

## Abbreviations

ANN: artificial neural network
DT: decision tree
ED: emergency department
FN: false negative
FP: false positive
GP: genetic programming
LR: logistic regression
MLPNN: multilayer perceptron neural network
RF: random forest
SVM: support vector machine
TN: true negative
TNR: true negative rate
TP: true positive
TPR: true positive rate
