# Supplementary material for: Predictive Modeling for Frailty Conditions in Elderly People: Machine Learning Approaches
Source: JMIR Med Inform. 2020 Jun 4;8(6):e16678. doi: 10.2196/16678 (PMC7303829; doi:10.2196/16678)
Supplement: Multimedia Appendix 1 [file medinform_v8i6e16678_app1.docx]

**Multimedia Appendix 1:**

Table 1.1 shows the description of 58 input variables used in the study. Table 1.2 presents the Chi-square test results between 10% and 90 % negative samples. Table 1.3 contains GP Control Parameters used in the Experiments.

Table 1.1 Description of input variables in the study

| Variable | Category | Code | Count | Percent (%) |
| --- | --- | --- | --- | --- |
| Age | 65-69 | 0 | 273,389 | 24.95 |
|  | 70-74 | 1 | 231,248 | 21.11 |
|  | 75-79 | 2 | 233,122 | 21.28 |
|  | 80-84 | 3 | 175,854 | 16.05 |
|  | 85-89 | 4 | 117,674 | 10.74 |
|  | >=90 | 5 | 64,326 | 5.87 |
| Citizenship | PSA* | 0 | 1,076,375 | 98.24 |
|  | PFPM** | 1 | 19,238 | 1.76 |
| Number of urgent hospitalization | 0 | 0 | 1,024,739 | 93.53 |
|  | 1 or 2 | 1 | 67,523 | 6.16 |
|  | 3 or more | 2 | 3,351 | 0.31 |
| Number of non-traumatic hospitalizations | 0 | 0 | 987,152 | 90.10 |
|  | 1 or 2 | 1 | 99,576 | 9.09 |
|  | 3 or more | 2 | 8,885 | 0.81 |
| Number of total hospitalizations | 0 | 0 | 977,028 | 89.18 |
|  | 1 or 2 | 1 | 107,881 | 9.85 |
|  | 3 or more | 2 | 10,704 | 0.98 |
| Charlson index (Charlson, Pompei, Ales, & MacKenzie, 1987) | Value index 0 | 0 | 1,019,406 | 93.04 |
|  | Index 1-2 | 1 | 61,717 | 5.63 |
|  | Index 3 - 5 | 2 | 10,160 | 0.93 |
|  | value index 6 or higher | 3 | 4,330 | 0.40 |
| Home-based care | no | 0 | 1,084,324 | 98.97 |
|  | yes | 1 | 11,289 | 1.03 |
| Income | no | 0 | 251,424 | 22.95 |
|  | yes | 1 | 844,189 | 77.05 |
| Invalidity | no | 0 | 959,791 | 87.60 |
|  | yes | 1 | 135,822 | 12.40 |
| Poly prescriptions  (number of drugs prescribed) | from 0 to 5 drugs | 0 | 559,731 | 51.09 |
|  | from 6 to 10 drugs | 1 | 366,355 | 33.44 |
|  | more than 11 drugs | 2 | 169,527 | 15.47 |
| Number of different types of drugs prescribed ( First three digits of ATC code) | 0 | 0 | 82,879 | 7.56 |
|  | from 1 to 5 | 1 | 566,187 | 51.68 |
|  | from 6 to 14 | 2 | 439,599 | 40.12 |
|  | 15 or more | 3 | 6,948 | 0.63 |
|  |  |  |  |  |
|  |  |  |  |  |
| emergency department visits with white code | no access | 0 | 1,062,489 | 96.98 |
|  | at least one access | 1 | 33,124 | 3.02 |
| emergency department visits with green code | no access | 0 | 879,634 | 80.29 |
|  | 1 or 2 accesses | 1 | 194,310 | 17.74 |
|  | 3 or more accesses | 2 | 21,669 | 1.98 |
| emergency department visits with yellow code | no access | 0 | 1,039,685 | 94.90 |
|  | 1 or 2 accesses | 1 | 54,426 | 4.97 |
|  | 3 or more accesses | 2 | 1,502 | 0.14 |
| emergency department visits with red code | no access | 0 | 1,092,082 | 99.68 |
|  | at least one access | 1 | 3,531 | 0.32 |
| Housing condition | privately owned | 1 | 890,222 | 81.25 |
|  | renting | 2 | 157,150 | 14.34 |
|  | other | 3 | 48,241 | 4.40 |
| Marital status | single | 1 | 81,492 | 7.44 |
|  | married | 2 | 697,589 | 63.67 |
|  | widower | 3 | 251,423 | 22.95 |
|  | divorced | 4 | 65,109 | 5.94 |
| Level of education | degree or superior average | 1 | 188,799 | 17.23 |
|  | lower average or professional qualification | 3 | 325,242 | 29.69 |
|  | elementary or without study title | 4 | 581,572 | 53.08 |
| Work status | employed | 1 | 95,188 | 8.69 |
|  | housewife | 3 | 102,523 | 9.36 |
|  | withdrawn from work | 5 | 851,202 | 77.69 |
|  | Other (unemployed, student, etc.) | 7 | 46,700 | 4.26 |
| Home living status | noncrowded | 1 | 1,020,696 | 93.16 |
|  | crowded | 2 | 74,917 | 6.84 |
| Type of family | an elderly couple (both over 65) without children | 1 | 375,189 | 34.24 |
|  | only with children | 2 | 63,561 | 5.80 |
|  | only without children, single or widower | 3 | 360,051 | 32.86 |
|  | other cohabitations | 4 | 296,812 | 27.09 |
| Disability | no | 0 | 950,048 | 86.71 |
|  | yes | 1 | 145,565 | 13.29 |
| Femur Fracture | no | 0 | 1,040,449 | 94.97 |
|  | yes | 1 | 55,164 | 5.04 |
| Depression | no | 0 | 929,835 | 84.87 |
|  | yes | 1 | 165,778 | 15.13 |
| Diabetes | no | 0 | 914,357 | 83.46 |
|  | yes | 1 | 181,256 | 16.54 |
| Arthropathy | no | 0 | 1,046,700 | 95.54 |
|  | yes | 1 | 48,913 | 4.46 |
| Parkinson's disease | no | 0 | 1,074,446 | 98.07 |
|  | yes | 1 | 21,167 | 1.93 |
| Epilepsy | no | 0 | 1,033,834 | 94.36 |
|  | yes | 1 | 61,779 | 5.64 |
| Anaemia | no | 0 | 1,018,977 | 93.01 |
|  | yes | 1 | 76,636 | 6.99 |
| Hypertensive | no | 0 | 782,954 | 71.46 |
|  | yes | 1 | 312,659 | 28.54 |
| Glaucoma | no | 0 | 1,016,227 | 92.75 |
|  | yes | 1 | 79,386 | 7.25 |
| Mental disease | no | 0 | 1,060,794 | 96.82 |
|  | yes | 1 | 34,819 | 3.18 |
| Cancer | no | 0 | 969,426 | 88.48 |
|  | yes | 1 | 126,187 | 11.52 |
| Thyroid | no | 0 | 992,007 | 90.54 |
|  | yes | 1 | 103,606 | 9.46 |
| Dementia | no | 0 | 1,080,388 | 98.61 |
|  | yes | 1 | 15,225 | 1.39 |
| Coronary artery disease | no | 0 | 1,048,234 | 95.68 |
|  | yes | 1 | 47,379 | 4.32 |
| Congestive heart failure | no | 0 | 1,082,722 | 98.82 |
|  | yes | 1 | 12,891 | 1.18 |
| Stroke | no | 0 | 1,067,443 | 97.43 |
|  | yes | 1 | 28,170 | 2.57 |
| Kidney failure | no | 0 | 1,084,182 | 98.96 |
|  | yes | 1 | 11,431 | 1.04 |
| Hypercholesterolemia | no | 0 | 1,067,678 | 97.45 |
|  | yes | 1 | 27,935 | 2.55 |
| Atrial fibrillation | no | 0 | 1,044,590 | 95.34 |
|  | yes | 1 | 51,023 | 4.66 |
| neck fracture | no | 0 | 1,084,155 | 98.95 |
|  | yes | 1 | 11,458 | 1.05 |
| Infectious diseases | no | 0 | 581,304 | 53.06 |
|  | yes | 1 | 514,309 | 46.94 |
| Neoplasia | no | 0 | 961,674 | 87.78 |
|  | yes | 1 | 133,939 | 12.23 |
| Blood disease | no | 0 | 659,726 | 60.22 |
|  | yes | 1 | 435,887 | 39.78 |
| Nerve disease | no | 0 | 698,471 | 63.75 |
|  | yes | 1 | 397,142 | 36.25 |
| Diseases of the respiratory system | no | 0 | 878,327 | 80.17 |
|  | yes | 1 | 217,286 | 19.83 |
| Muscular diseases | no | 0 | 110,723 | 10.11 |
|  | yes | 1 | 984,890 | 89.89 |
| Diseases of the urinary tract | no | 0 | 1,034,044 | 94.38 |
|  | yes | 1 | 61,569 | 5.62 |
| Diseases of the digestive tract | no | 0 | 1,035,118 | 94.48 |
|  | yes | 1 | 60,495 | 5.52 |
| Endocrine diseases | no | 0 | 871,693 | 79.56 |
|  | yes | 1 | 223,920 | 20.44 |
| diseases of the circulatory system | no | 0 | 683,534 | 62.39 |
|  | yes | 1 | 412,079 | 37.61 |
| Metabolism diseases | no | 0 | 431,410 | 39.38 |
|  | yes | 1 | 664,203 | 60.62 |
| Cardiovascular diseases | no | 0 | 299,798 | 27.36 |
|  | yes | 1 | 795,815 | 72.64 |
| Drugs for dermatological problems | no | 0 | 1,068,253 | 97.50 |
|  | yes | 1 | 27,360 | 2.50 |
| Genital diseases | no | 0 | 946,439 | 86.38 |
|  | yes | 1 | 149,174 | 13.62 |
| Hormonal diseases | no | 0 | 839,661 | 76.64 |
|  | yes | 1 | 255,952 | 23.36 |
| Problems with the sensory parts | no | 0 | 1,019,402 | 93.04 |
|  | yes | 1 | 76,211 | 6.96 |

* People with citizenship from advanced development countries

**People with citizenship in countries with high migratory pressure

Table 1.2. Chi-square test results between 10% and 90 % negative samples.

| **Variable Name** | **Code** | **Sample-1 (10%)** | | **Sample-2 (90%)** | | **X^2^** | **DF** | **CV** | ***P-*values** |
| --- | --- | --- | --- | --- | --- | --- | --- | --- | --- |
|  |  | **Count** | **%** | **N** | **%** |  |  |  |  |
| Age | 0 | 27,897 | 26 | 234,694 | 27 | 4.36 | 5 | 11.07 | .50 |
|  | 1 | 23,512 | 22 | 195,078 | 22 |  |  |  |  |
|  | 2 | 22,912 | 22 | 191,267 | 22 |  |  |  |  |
|  | 3 | 16,560 | 16 | 136,996 | 16 |  |  |  |  |
|  | 4 | 10,113 | 10 | 84,746 | 10 |  |  |  |  |
|  | 5 | 4,968 | 5 | 40,908 | 5 |  |  |  |  |
| Citizenship | 0 | 104,088 | 98 | 867,665 | 98 | 1.07 | 1 | 3.84 | .30 |
|  | 1 | 1,874 | 2 | 16,024 | 2 |  |  |  |  |
| Number of urgent hospitalization | 0 | 100,886 | 95 | 840,603 | 95 | 1.90 | 2 | 5.99 | .39 |
|  | 1 | 4,931 | 5 | 41,785 | 5 |  |  |  |  |
|  | 2 | 145 | 0 | 1,301 | 0 |  |  |  |  |
| Number of nontraumatic hospitalizations | 0 | 97,230 | 92 | 809,990 | 92 | 2.65 | 2 | 5.99 | .27 |
|  | 1 | 8,149 | 8 | 69,023 | 8 |  |  |  |  |
|  | 2 | 583 | 1 | 4,676 | 1 |  |  |  |  |
| Number of total hospitalizations | 0 | 96,423 | 91 | 803,210 | 91 | 1.86 | 2 | 5.99 | .39 |
|  | 1 | 8,840 | 8 | 74,757 | 8 |  |  |  |  |
|  | 2 | 699 | 1 | 5,722 | 1 |  |  |  |  |
| Charlson index | 0 | 100,247 | 95 | 836,048 | 95 | 0.69 | 3 | 7.81 | .88 |
|  | 1 | 4,836 | 5 | 40,120 | 5 |  |  |  |  |
|  | 2 | 627 | 1 | 5,392 | 1 |  |  |  |  |
|  | 3 | 252 | 0 | 2,129 | 0 |  |  |  |  |
| Home-based care | 0 | 104,868 | 99 | 874,682 | 99 | 0.16 | 1 | 3.84 | .69 |
|  | 1 | 1,094 | 1 | 9,007 | 1 |  |  |  |  |
| Income | 0 | 24,990 | 24 | 208,621 | 24 | 0.03 | 1 | 3.84 | .86 |
|  | 1 | 80,972 | 76 | 675,068 | 76 |  |  |  |  |
| Invalidity | 0 | 94,608 | 89 | 789,101 | 89 | 0.01 | 1 | 3.84 | .91 |
|  | 1 | 11,354 | 11 | 94,588 | 11 |  |  |  |  |
| Poly prescriptions  (number of drugs prescribed) | 0 | 56,475 | 53 | 470,901 | 53 | 2.50 | 2 | 5.99 | .29 |
|  | 1 | 35,262 | 33 | 292,723 | 33 |  |  |  |  |
|  | 2 | 14,225 | 13 | 120,065 | 14 |  |  |  |  |
| Number of different types of drugs prescribed ( First three digits of ATC code) | 0 | 8,573 | 8 | 70,642 | 8 | 1.25 | 3 | 7.81 | .74 |
|  | 1 | 56,665 | 53 | 472,961 | 54 |  |  |  |  |
|  | 2 | 40,254 | 38 | 336,201 | 38 |  |  |  |  |
|  | 3 | 470 | 0 | 3,885 | 0 |  |  |  |  |
| Access to ED with White code | 0 | 102,833 | 97 | 857,402 | 97 | 0.16 | 1 | 3.84 | .69 |
|  | 1 | 3,129 | 3 | 26,287 | 3 |  |  |  |  |
| Access to ED with Green code | 0 | 86,958 | 82 | 724,742 | 82 | 0.34 | 2 | 5.99 | .84 |
|  | 1 | 17,399 | 16 | 145,384 | 16 |  |  |  |  |
|  | 2 | 1,605 | 2 | 13,563 | 2 |  |  |  |  |
| Access to ED with Yellow code | 0 | 101,762 | 96 | 847,565 | 96 | 3.82 | 2 | 5.99 | .15 |
|  | 1 | 4,122 | 4 | 35,477 | 4 |  |  |  |  |
|  | 2 | 78 | 0 | 647 | 0 |  |  |  |  |
| Access to ED with Red code | 0 | 105,740 | 100 | 881,738 | 100 | 0.55 | 1 | 3.84 | .46 |
|  | 1 | 222 | 0 | 1,951 | 0 |  |  |  |  |
| Housing condition | 1 | 86,678 | 82 | 722,002 |  | 0.55 | 2 | 5.99 | .76 |
|  | 2 | 14,853 | 14 | 123,963 | 14 |  |  |  |  |
|  | 3 | 4,431 | 4 | 37,724 | 4 |  |  |  |  |
| Marital status | 1 | 7,730 | 7 | 65,119 | 7 | 1.10 | 3 | 7.81 | .78 |
|  | 2 | 68,656 | 65 | 572,836 | 65 |  |  |  |  |
|  | 3 | 23,153 | 22 | 192,280 | 22 |  |  |  |  |
|  | 4 | 6,423 | 6 | 53,454 | 6 |  |  |  |  |
| Level of education | 1 | 18,736 | 18 | 157,134 | 18 | 0.75 | 2 | 5.99 | .69 |
|  | 3 | 32,145 | 30 | 268,151 | 30 |  |  |  |  |
|  | 4 | 55,081 | 52 | 458,404 | 52 |  |  |  |  |
| Work status | 1 | 9,680 | 9 | 80,955 | 9 | 0.29 | 3 | 7.81 | .96 |
|  | 3 | 10,155 | 10 | 84,838 | 10 |  |  |  |  |
|  | 5 | 81,694 | 77 | 680,710 | 77 |  |  |  |  |
|  | 7 | 4,433 | 4 | 37,186 | 4 |  |  |  |  |
| Home living status | 1 | 98,811 | 93 | 823,362 | 93 | 0.90 | 1 | 3.84 | .34 |
|  | 2 | 7,151 | 7 | 60,327 | 7 |  |  |  |  |
| Type of family | 1 | 36,780 | 35 | 305,745 | 35 | 1.66 | 3 | 7.81 | .65 |
|  | 2 | 6,147 | 6 | 50,654 | 6 |  |  |  |  |
|  | 3 | 33,482 | 32 | 280,202 | 32 |  |  |  |  |
|  | 4 | 29,553 | 28 | 247,088 | 28 |  |  |  |  |
| Disability | 0 | 93,636 | 88 | 781,148 | 88 | 0.08 | 1 | 3.84 | .78 |
|  | 1 | 12,326 | 12 | 102,541 | 12 |  |  |  |  |
| Femur Fracture | 0 | 101,331 | 96 | 843,904 | 96 | 3.83 | 1 | 3.84 | .05 |
|  | 1 | 4,631 | 4 | 39,785 | 5 |  |  |  |  |
| Depression | 0 | 91,148 | 86 | 759,378 | 86 | 0.59 | 1 | 3.84 | .44 |
|  | 1 | 14,814 | 14 | 124,311 | 14 |  |  |  |  |
| Diabetes | 0 | 89,437 | 84 | 744,236 | 84 | 2.45 | 1 | 3.84 | .12 |
|  | 1 | 16,525 | 16 | 139,453 | 16 |  |  |  |  |
| Arthropathy | 0 | 101,320 | 96 | 845,264 | 96 | 0.24 | 1 | 3.84 | .62 |
|  | 1 | 4,642 | 4 | 38,425 | 4 |  |  |  |  |
| Parkinson's disease | 0 | 104,233 | 98 | 869,700 | 98 | 1.44 | 1 | 3.84 | .23 |
|  | 1 | 1,729 | 2 | 13,989 | 2 |  |  |  |  |
| Epilepsy | 0 | 100,577 | 95 | 838,499 | 95 | 0.20 | 1 | 3.84 | .66 |
|  | 1 | 5,385 | 5 | 45,190 | 5 |  |  |  |  |
| Anemia | 0 | 99,657 | 94 | 830,663 | 94 | 0.42 | 1 | 3.84 | .51 |
|  | 1 | 6,305 | 6 | 53,026 | 6 |  |  |  |  |
| Hypertensive | 0 | 76,533 | 72 | 637,821 | 72 | 0.12 | 1 | 3.84 | .73 |
|  | 1 | 29,429 | 28 | 245,868 | 28 |  |  |  |  |
| Glaucoma | 0 | 98,385 | 93 | 820,530 | 93 | 0.00 | 1 | 3.84 | .97 |
|  | 1 | 7,577 | 7 | 63,159 | 7 |  |  |  |  |
| Mental disease | 0 | 103,095 | 97 | 860,158 | 97 | 0.67 | 1 | 3.84 | .41 |
|  | 1 | 2,867 | 3 | 23,531 | 3 |  |  |  |  |
| Cancer | 0 | 94,429 | 89 | 787,377 | 89 | 0.02 | 1 | 3.84 | .88 |
|  | 1 | 11,533 | 11 | 96,312 | 11 |  |  |  |  |
| Thyroid | 0 | 96,048 | 91 | 801,056 | 91 | 0.00 | 1 | 3.84 | .96 |
|  | 1 | 9,914 | 9 | 82,633 | 9 |  |  |  |  |
| Dementia | 0 | 104,948 | 99 | 875,171 | 99 | 0.05 | 1 | 3.84 | .83 |
|  | 1 | 1,014 | 1 | 8,518 | 1 |  |  |  |  |
| Coronary artery disease | 0 | 101,713 | 96 | 848,154 | 96 | 0.03 | 1 | 3.84 | .86 |
|  | 1 | 4,249 | 4 | 35,535 | 4 |  |  |  |  |
| Congestive heart failure | 0 | 105,099 | 99 | 876,572 | 99 | 0.10 | 1 | 3.84 | .76 |
|  | 1 | 863 | 1 | 7,117 | 1 |  |  |  |  |
| Stroke | 0 | 103,549 | 98 | 863,804 | 98 | 0.31 | 1 | 3.84 | .58 |
|  | 1 | 2,413 | 2 | 19,885 | 2 |  |  |  |  |
| kidney failure | 0 | 105,048 | 99 | 875,966 | 99 | 0.14 | 1 | 3.84 | .71 |
|  | 1 | 914 | 1 | 7,723 | 1 |  |  |  |  |
| Hypercholesterolemia | 0 | 103,254 | 97 | 861,238 | 97 | 0.09 | 1 | 3.84 | .77 |
|  | 1 | 2,708 | 3 | 22,451 | 3 |  |  |  |  |
| Atrial fibrillation | 0 | 101,702 | 96 | 848,265 | 96 | 0.03 | 1 | 3.84 | .86 |
|  | 1 | 4,260 | 4 | 35,424 | 4 |  |  |  |  |
| Neck fracture | 0 | 105,117 | 99 | 876,678 | 99 | 0.02 | 1 | 3.84 | .89 |
|  | 1 | 845 | 1 | 7,011 | 1 |  |  |  |  |
| Infectious diseases | 0 | 57,484 | 54 | 478,823 | 54 | 0.16 | 1 | 3.84 | .69 |
|  | 1 | 48,478 | 46 | 404,866 | 46 |  |  |  |  |
| Neoplasia | 0 | 93,736 | 88 | 781,662 | 88 | 0.01 | 1 | 3.84 | .94 |
|  | 1 | 12,226 | 12 | 102,027 | 12 |  |  |  |  |
| Blood disease | 0 | 66,208 | 62 | 553,085 | 63 | 0.45 | 1 | 3.84 | .50 |
|  | 1 | 39,754 | 38 | 330,604 | 37 |  |  |  |  |
| Nerve disease | 0 | 69,698 | 66 | 580,218 | 66 | 0.58 | 1 | 3.84 | .45 |
|  | 1 | 36,264 | 34 | 303,471 | 34 |  |  |  |  |
| Diseases of the respiratory system | 0 | 86,452 | 82 | 720,816 | 82 | 0.02 | 1 | 3.84 | .88 |
|  | 1 | 19,510 | 18 | 162,873 | 18 |  |  |  |  |
| Muscular diseases | 0 | 9,649 | 9 | 80,108 | 9 | 0.19 | 1 | 3.84 | .66 |
|  | 1 | 96,313 | 91 | 803,581 | 91 |  |  |  |  |
| Diseases of the urinary tract | 0 | 100,843 | 95 | 841,242 | 95 | 0.16 | 1 | 3.84 | 0.69 |
|  | 1 | 5,119 | 5 | 42,447 | 5 |  |  |  |  |
| Diseases of the digestive tract | 0 | 100,932 | 95 | 840,559 | 95 | 3.65 | 1 | 3.84 | .06 |
|  | 1 | 5,030 | 5 | 43,130 | 5 |  |  |  |  |
| Endocrine diseases | 0 | 85,300 | 81 | 710,744 | 80 | 0.31 | 1 | 3.84 | .58 |
|  | 1 | 20,662 | 20 | 172,945 | 20 |  |  |  |  |
| Diseases of the circulatory system | 0 | 68,293 | 64 | 569,475 | 64 | 0.00 | 1 | 3.84 | .96 |
|  | 1 | 37,669 | 36 | 314,214 | 36 |  |  |  |  |
| Metabolism diseases | 0 | 43,419 | 41 | 360,291 | 41 | 1.64 | 1 | 3.84 | .20 |
|  | 1 | 62,543 | 59 | 523,398 | 59 |  |  |  |  |
| Cardiovascular diseases | 0 | 30,111 | 28 | 250,030 | 28 | 0.70 | 1 | 3.84 | .40 |
|  | 1 | 75,851 | 72 | 633,659 | 72 |  |  |  |  |
| Drugs for dermatological problems | 0 | 103,420 | 98 | 862,639 | 98 | 0.12 | 1 | 3.84 | .73 |
|  | 1 | 2,542 | 2 | 21,050 | 2 |  |  |  |  |
| Genital diseases | 0 | 91,741 | 87 | 765,505 | 87 | 0.18 | 1 | 3.84 | .67 |
|  | 1 | 14,221 | 13 | 118,184 | 13 |  |  |  |  |
| Hormonal diseases | 0 | 82,094 | 77 | 683,505 | 77 | 0.89 | 1 | 3.84 | .35 |
|  | 1 | 23,868 | 23 | 200,184 | 23 |  |  |  |  |
| Problems with the sensory parts | 0 | 98,687 | 93 | 822,827 | 93 | 0.07 | 1 | 3.84 | .79 |
|  | 1 | 7,275 | 7 | 60,862 | 7 |  |  |  |  |
