# Supplementary material for: Predictive Modeling for Frailty Conditions in Elderly People: Machine Learning Approaches
Source: JMIR Med Inform. 2020 Jun 4;8(6):e16678. doi: 10.2196/16678 (PMC7303829; doi:10.2196/16678)
Supplement: Multimedia Appendix 2 [file medinform_v8i6e16678_app2.docx]

**Multimedia Appendix 2:**

2.1 Parameters configurations for each machine learning model used in the experiment.

| **HyperParameters settings for each ML model in each of the six problems** | | | | | |
| --- | --- | --- | --- | --- | --- |
| **Problem** | SVM | ANN | RF | DT | LR |
| Mortality | 'C': 100, 'gamma': 0.001, 'kernel': 'rbf' | 'activation': 'relu', 'alpha': 0.05, 'hidden_layer_sizes': (30, 30, 30), 'learning_rate': 'constant', 'solver': 'sgd' | 'max_depth': 90, 'max_features': 3, 'min_samples_leaf': 5, 'min_samples_split': 12, 'n_estimators': 100 | 'criterion': 'entropy', 'max_depth': 12, 'max_features': 5, 'min_samples_split': 2 | 'C': 10.0, 'penalty': 'l2' |
| Fracture | 'C': 1, 'gamma': 0.01, 'kernel': 'rbf' | 'activation': 'relu', 'alpha': 0.05, 'hidden_layer_sizes': (50, 100, 50), 'learning_rate': 'adaptive', 'solver': 'sgd' | 'max_depth': 80, 'max_features': 8, 'min_samples_leaf': 5, 'min_samples_split': 12, 'n_estimators': 200 | 'criterion':'entropy', 'max_depth': 6, 'max_features': 30, 'min_samples_split': 5 | 'C': 1000.0, 'penalty': 'l2' |
| Disability | 'C': 1, 'gamma': 0.1, 'kernel': 'rbf' | 'activation': 'tanh', 'alpha': 0.05, 'learning_rate': 'constant', 'solver':‘sgd,hidden_layer_sizes':(10,10,10) | 'max_features': 6, 'min_samples_leaf': 3, 'min_samples_split': 8, 'n_estimators': 100, ‘max_depth':80 | 'max_depth': 100, 'max_features': 3, 'min_samples_split': 12, 'n_estimators': 100 | 'C': 100.0, 'penalty': 'l2' |
| Urgent Hosp. | 'C': 100, 'gamma': 0.001, 'kernel': 'rbf' | 'activation': 'relu', 'alpha': 0.0001, 'learning_rate': 'constant', 'solver':‘adam,hidden_layer_sizes':(100,) | 'max_features': 3, 'min_samples_leaf': 5, 'min_samples_split': 12, 'n_estimators': 100, ‘max_depth':90 | 'criterion': 'entropy', 'max_depth': 6, 'max_features': 30, 'min_samples_split': 2 | 'C': 1.0, 'penalty': 'l2' |
| Preventable Hosp. | 'C': 10, 'gamma': 0.001, 'kernel': 'rbf' | 'activation': 'tanh', 'alpha': 0.05, 'hidden_layer_sizes': (100,), 'learning_rate': 'constant', 'solver': 'sgd' | 'max_features': 3, 'min_samples_leaf': 4, 'min_samples_split': 12, 'n_estimators': 300, ‘max_depth':90 | 'criterion': 'gini', 'max_depth': 6, 'max_features': 10, 'min_samples_split': 2 | 'C': 10.0, 'penalty': 'l2' |
| Access to emergency department with red code | 'C': 1, 'gamma': 0.1, 'kernel': 'rbf' | 'activation': 'relu', 'alpha': 0.0001, 'learning_rate': 'adaptive', 'solver': 'sgd' | 'max_depth': 80, 'max_features': 10, 'min_samples_leaf': 4, 'min_samples_split': 2, 'n_estimators': 300 | 'criterion': 'entropy', 'max_depth': 6, 'max_features': 40, 'min_samples_split': 2 | 'C': 0.1, 'penalty': 'l2' |

2.2. GP Control Parameters used in the Experiment

| **Parameter** | **Value** |
| --- | --- |
| Population size | 1,000 |
| Maximum number of generations | 100 |
| Crossover probability | 0.9 |
| Mutation probability | 0.15 |
| Selection method | Tournament selection |
| Termination condition | Maximum generation |
| Solution creator | Probabilistic tree creator |
| Genetic operators | Crossover, Mutation |
| Elites | 1 |
| Maximum tree depth | 10 |
| Maximum tree length | 100 |
