# Supplementary material for: Predictive Modeling for Frailty Conditions in Elderly People: Machine Learning Approaches
Source: JMIR Med Inform. 2020 Jun 4;8(6):e16678. doi: 10.2196/16678 (PMC7303829; doi:10.2196/16678)
Supplement: Multimedia Appendix 4 [file medinform_v8i6e16678_app4.docx]

**Multimedia Appendix 4:**

Some of the most important features in each outcome

| **Disability problem** | | | **Urgent hospitalization problem** | | |
| --- | --- | --- | --- | --- | --- |
| **Variable** | **Rank** | ***P*-Value** | **Variable** | **Rank** | ***P*-Value** |
| Age | 1 | *P*<.001 | Age | 1 | *P*<.001 |
| Charlson index | 2 | *P*<.001 | Mental Disease | 2 | *P*<.001 |
| # total hospitalizations | 3 | *P*<.001 | Poly prescriptions | 3 | *P*<.001 |
| # urgent hospitalization | 4 | *P*<.001 | diseases of the respiratory system | 4 | *P*<.001 |
| Poly prescriptions | 5 | *P*<.001 | Citizenship | 5 | *P*<.001 |
| # non-traumatic | 6 | *P*<.001 | White code | 6 | *P*<.001 |
| Green code | 7 | *P*<.001 | arthropathy | 7 | *P*<.001 |
| Nerve disease | 8 | *P*<.001 | diseases of the circulatory system | 8 | *P*<.001 |
| Disability | 10 | *P*<.001 | Glaucoma | 10 | *P*<.001 |
| blood disease | 11 | *P*<.001 | Femur fracture | 11 | *P*<.001 |
| Yellow code | 12 | *P*<.001 | Heart disease | 12 | *P*<.001 |
| Depression | 13 | *P*<.001 | Nerve disease | 13 | *P*<.001 |
| diseases of the circulatory system | 14 | *P*<.001 | Neoplasm | 14 | *P*<.001 |
| Dementia | 15 | *P*<.001 | Disability | 15 | *P*<.001 |
| Anaemia | 16 | *P*<.001 | Drugs for dermatological problems | 16 | *P*<.001 |
| mental disease | 17 | *P*<.001 | metabolism diseases | 17 | *P*<.001 |
| diseases of the urinary tract | 18 | *P*<.001 | genital diseases | 18 | *P*<.001 |
| Parkinson disease | 19 | *P*<.001 | hormonal diseases | 19 | *P*<.001 |

Table 4.1. The most important variables in disability and urgent hospitalization problems

4.2. The most important variables in preventable hospitalization and emergency admission.

| **Preventable hospitalization** | | | **Emergency admission with red code** | | |
| --- | --- | --- | --- | --- | --- |
| **Variable** | **Rank** | ***P*-Value** | **Variable** | **Rank** | ***P*-Value** |
| Age | 1 | *P*<.001 | Age | 1 | *P*<.001 |
| Mental disease | 2 | *P*<.001 | Charlson index | 2 | *P*<.001 |
| Poly prescriptions | 3 | *P*<.001 | # urgent hospitalization | 3 | *P*<.001 |
| diseases of the respiratory system | 4 | *P*<.001 | # total hospitalization | 4 | *P*<.001 |
| White code | 5 | *P*<.001 | Poly prescriptions | 5 | *P*<.001 |
| Citizenship | 6 | *P*<.001 | # non-traumatic | 6 | *P*<.001 |
| arthropathy | 7 | *P*<.001 | Yellow code | 7 | *P*<.001 |
| diseases of the circulatory system | 8 | *P*<.001 | Invalidity | 8 | *P*<.001 |
| Glaucoma | 10 | *P*<.001 | Disability | 10 | *P*<.001 |
| Heat disease | 12 | *P*<.001 | diseases of the respiratory system | 12 | *P*<.001 |
| Femur fracture | 13 | *P*<.001 | Blood disease | 13 | *P*<.001 |
| Nerve disease | 14 | *P*<.001 | diseases of the circulatory system | 14 | *P*<.001 |
| Neoplasm | 15 | *P*<.001 | Green code | 15 | *P*<.001 |
| metabolism diseases | 16 | *P*<.001 | diseases of the urinary tract | 16 | *P*<.001 |
| Drugs for dermatological problems | 17 | *P*<.001 | Anaemia | 17 | *P*<.001 |
| drugs for the sensory parts | 18 | *P*<.001 | Congestive heart failure | 18 | *P*<.001 |
